# Effect of degree of urbanisation on age and sex-specific asthma prevalence in Swedish preschool children

**DOI:** 10.1186/1471-2458-9-303

**Published:** 2009-08-20

**Authors:** Kristina Bröms, Dan Norbäck, Margaretha Eriksson, Claes Sundelin, Kurt Svärdsudd

**Affiliations:** 1 Department of Public Health and Caring Sciences, Family Medicine and Clinical Epidemiology, Uppsala University, Uppsala, Sweden; 2 Centre for Clinical Research Uppsala University, County Council of Gävleborg, Gävle, Sweden; 3 Department of Medical Sciences, Occupational and Environmental Medicine, Uppsala University, Uppsala, Sweden; 4 Department of Women's and Children's Health, Paediatrics, Uppsala University, Uppsala, Sweden

## Abstract

**Background:**

There are few studies on age and sex-specific asthma prevalence in the age range 1–6 years. The purpose of this report was to estimate age and sex specific asthma prevalence in preschool children and to analyse the influence of possible demographic and geographic determinants.

**Methods:**

All 70 allergen avoidance day-care centres and 140 matched ordinary day-care centres across Sweden were sampled. The parents of all 8,757 children attending these day-care centres received the International Study of Asthma and Allergies in Childhood (ISAAC) written questionnaire, supplemented with questions on medical treatment, physician assessed asthma diagnosis, and other asthma related questions. The response rate was 68%.

**Results:**

The age specific asthma prevalence, adjusted for the underlying municipality population size, was among boys 9.7% at age 1, 11.1% at age 2, 11.4 at age 3, 10.5 at age 4, 8.7 at age 5, and 6.4 at age 6. The corresponding proportions among girls were 8.9%, 9.9%, 9.8%, 8.8%, 7.0%, and 5.0%, on average 9.6% for boys and 8.2% for girls, altogether 8.9%. In addition to age and sex the prevalence increased by municipality population density, a proxy for degree of urbanisation. Moreover, there was a remaining weak geographical gradient with increasing prevalence towards the north and the west.

**Conclusion:**

The age-specific asthma prevalence was curvilinear with a peak around age 3 and somewhat higher for boys than for girls. The asthma prevalence increased in a slowly accelerating pace by municipality population density as a proxy for degree of urbanisation.

## Background

In recent decades, the prevalence of allergies and asthma in industrialized countries has increased, particularly among children, but during the last few years the increase rate may have diminished [1-3]. According to studies made by the International Study of Asthma and Allergies in Childhood (ISAAC), the prevalence of wheezing during the last year among 6–7 year old children in Sweden is in the middle of the international range [3-5].

However, there are few published studies on age and sex-specific asthma prevalence in the age range 1–6 years [6-10]. Geographical prevalence differences have been found in the ISAAC studies, with the highest levels in English speaking countries and Latin America, and low levels in Africa and Asia except Singapore and Japan [3,11]. In Europe there is a northwest-southeast gradient with high levels in the northwest [3], although other researchers found no gradients in Western Europe [12].

We found only one study on possible gradients within countries showing an increasing prevalence towards the north among Swedish school children [13]. However, it is doubtful what geographical gradients stand for. Possible explanations might be degree of urbanisation or climate. A number of studies have analysed differences in asthma prevalence between rural and urban areas with conflicting results [14-19]. However, as far as we know there are no studies on how much of the influence of geographical location on asthma prevalence that can be explained by degree of urbanisation.

This study was performed within the framework of a large-scale Swedish longitudinal project with nationwide coverage and with the main aim to study effects on asthma and allergy symptoms of low allergen exposure versus normal exposure in preschool children. The aims of this report, based on baseline data in the project, were to study the effects on asthma prevalence of various diagnostic criteria, to estimate age and sex-specific asthma prevalence in preschool children, and to study possible effects on asthma prevalence of geographical location (latitude and longitude) and municipality population size and density as proxies of degree of urbanisation.

## Methods

### Setting

Sweden is one of the most sparsely populated areas in Europe with a total population of 9 million dispersed over an area of 450,000 square kilometres. The median population density is 26 persons per square kilometre, and 80% of municipalities have 82 persons or less per square kilometre. The corresponding numbers for the municipalities included in this study were 57 and 130 persons, respectively.

**Figure 1:**
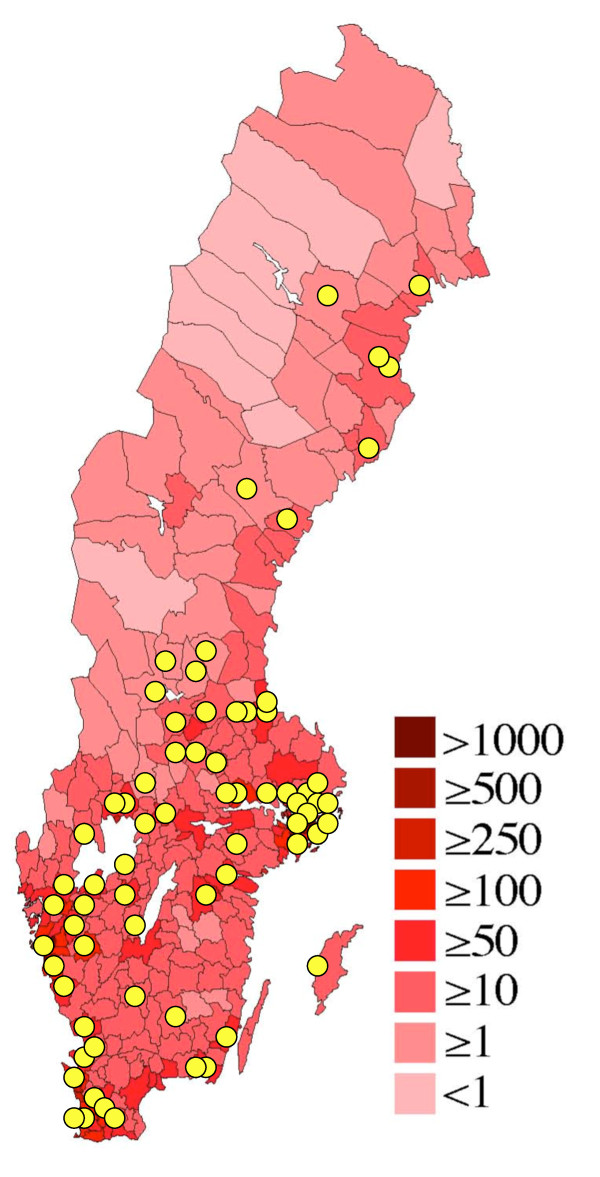
**Location of study population**. Map showing population density in Sweden by municipality, and localisation of the study day care centres (yellow circles). Modified from

For administrative purposes the country at the time of the data collection was divided into 25 regions and 290 municipalities, the smallest administrative unit. The distribution of mean population density by municipality is shown in Figure 1.

All Swedish preschool children are by law entitled to day-care organised by the local municipality. In 2002, 74% of all preschool children attended a day-care centre (DC), somewhat lower among the youngest children and more than 80% among children 3 years or older [20]. The vast majority of DCs are run by the local municipality administration. The few privately operated DCs are all subcontractors to the municipality administration and follow the same set of rules as publicly operated DCs. A DC may have one to four sections. At the time of the study 15–20 children were cared for in each section. Many sections had children of any age, but some were age stratified (1–3 years or 4–6 years). The day care fees are heavily subsidised by the municipalities, parents usually pay about 10% of the real cost.

Parents who want a position for their child in a DC have to file an application. Only children living in the municipality are accepted. If so, the child is put on a municipality administration operated waiting list, common for all DCs in the municipality. As soon as a position becomes vacant the parents of the child next in turn on the waiting list is offered the position. If the parents do not accept the offered position, the child may stay on the waiting list awaiting a position at their favourite DC, but in most instances this is impractical, since the waiting time for a specific DC may be more than a year, and private day care outside the municipality system is not subsidised.

During the 1990s special DCs for children with asthma or allergies (AADCs) were established. Municipal school administrations, parents, local politicians, and local DC staff took the initiative. The operations, set of rules, and fees for these centres are the same as for ordinary day care centres (ODC) with the exception that AADCs have a priority for children with asthma or allergies, but accept other children as well, space permitting. As shown in a previous report all AADCs had strict regulations to avoid pet, smoking, perfume, and dust exposure [21]. The ODCs usually had no such regulations.

### Study population

In the late 1990s all 72 AADCs in Sweden were identified. The two geographically closest ODCs to each AADC were chosen as control centres. Later, a few AADCs were closed and a few new were opened, leaving 70 AADCs with 84 sections and 140 ODCs with 440 sections for this study, in 62 municipalities, covering all of Sweden, Figure 1. One third of the AADCs were located in the same building as a control ODC.

The addresses of the 1,412 children attending the AADCs and the 7,345 children attending the ODCs were obtained from the local school authorities. A questionnaire was mailed to the parents of these children. Responses regarding 1,001 AADC children (70.9%) and 4958 ODC children (67.5%) were obtained after two reminders when necessary. Of the respondents, 1000 AADC children and 4,886 ODC children were 6 years or younger. They constitute the study population for this report.

### Data collection

The ISAAC written screening questionnaire with questions about asthma and wheezing, eczema and rhinitis, extensively used all over the world and regarded as a gold standard for postal questionnaires on childhood asthma, was used. Even though intended for children 6 years or older it has been validated down to three years of age with good results [22]. For this study, supplementary questions on medical treatment, physician assessed asthma diagnosis, parental education, smoking habits, and some additional variables not used here, were added.

Data on the number of boys and girls per one-year age groups in the age range 1–6 years in 2002 for each of the 62 municipalities was downloaded from Statistics Sweden [23], as were data on municipality total population size, population density (population per square kilometre municipality area), and national population distribution in the three traditional parts of Sweden (Götaland, Svealand, and Norrland). Information on geographical coordinates (latitude and longitude) of the municipalities was obtained from the National Land Survey of Sweden. The latitude range was 55.6–65.7 degrees North and the longitude range was 11.9–22.0 degrees East.

The study was approved on several occasions before and during the data collection process, first by the Research Ethics Committee at Uppsala University and later by the National Research Ethics Board.

### Statistical considerations

The statistical analyses were conducted using the SAS software [24]. Partial non-response (missing data in returned questionnaires) was on average 0.6% with a maximum in individual variables of 1.1%. Summary statistics such as means and measures of dispersion were computed using standard parametric methods. Simple differences between groups in proportions were tested with the chi-square test.

Two models for the prevalence calculations were used. In the first model, often used in other similar studies, only the ODC study population data was used, on the assumption that it represents a random sample of Swedish preschool children. However, this assumption may be questioned since most of the municipalities were represented in this study by only one AADC and two ODCs, irrespective of population size, causing an under-representation of large municipalities in the calculations of asthma prevalence.

Therefore, a second model was employed in which the number of children with asthma by age and sex and the total number of children by age and sex in each municipality was obtained. The number of children with asthma in each age and sex group was calculated as: (% children with asthma in the local ODCs) × (number of children in the municipality) + number of children with asthma in the local AADCs. The nationwide age and sex specific asthma prevalence was obtained as the ratio of the total number of children with asthma across all municipalities and the total number of children across all municipalities, thereby automatically weighted for municipality size.

The analyses of asthma prevalence determinants were performed with logistic regression using asthma diagnosis (model 1) or asthma prevalence (model 2) as the dependent variable and age, sex and other possible determinants as independent variables, providing odds ratios and their confidence intervals, p-values, and Wald's chi-square. The latter is the so far best measure of independent variable impact on the dependent variable.

As shown in Table 1, the number of one-year-old children was smaller than in the other age groups. However, the results in specific age-sex groups were based on estimates from the full model, which makes small numbers in certain subgroups of less importance. Moreover, age-sex specific confidence intervals are provided for the main results.

**Table 1:** Characteristics : Characteristics of the study population.

	Asthma-allergy day-care centres		Ordinary day-care centres	
	
	n	%	n	%
Age	1000	-	4886	-
1	49	4.9	203	4.2
2	219	21.9	916	18.8
3	221	22.1	976	20.0
4	192	19.2	1017	20.8
5	202	20.2	1168	23.9
6	116	11.6	606	12.4
Boys	545	54.5	2476	50.7
Parts of Sweden				
South (Götaland)	416	41.6	2135	43.7
Central (Svealand)	394	39.4	1841	37.7
North (Norrland)	190	19.0	910	18.6

**Figure 2:**
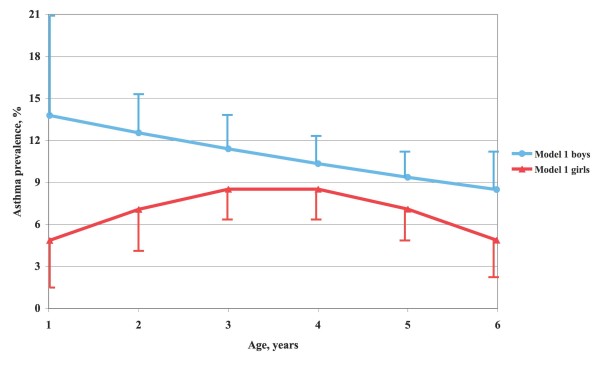
**Asthma prevalence by age and sex according to model 1**. Asthma prevalence (%) in Swedish preschool children by age and sex, based on the day care centre study population data (model 1).

**Figure 3:**
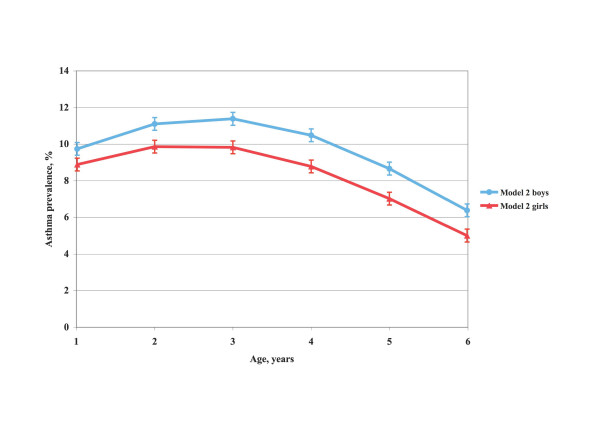
**Asthma prevalence by age and sex according to model 2**. Asthma prevalence (%) in Swedish preschool children by age and sex, based on calculations of the proportion of children with asthma in the total child population, 1–6 years of age, in the municipalities covered by the study (model 2).

The fit between the crude age and sex-specific prevalence and that obtained from the two analysis models was tested with logistic regression technique. In model 2, inclusion of age, age squared and an interaction term between age and sex as independent variables gave the best fit, explaining 50% of the prevalence variation. In model 1, the prevalence across age appeared to be linear for boys, whereas that for girls was similar to the trend line in model 2. On scrutiny, the fits appeared excellent. The curves in Figures 2 and 3 were obtained with logistic regression technique. All tests were two-tailed. The level of significance was set at p < 0.05.

## Results

### Characteristics of the study population

The proportion of children was similar in the age range 2–5 years and lower for those 1 and 6 years old, Table 1. Boys made up slightly more than half of the study population. The regional distribution was approximately the same as that of the Swedish national population. The age, sex and regional distributions were similar in the AADCs and ODCs.

### Questionnaire responses

As expected, the children in the AADCs reported more symptoms and higher rates of asthma than children in the ODCs, Table 2. The most frequently reported symptoms in the total study population were ever wheezing, wheezing any time during the last 12 months, dry cough at night with no cold, and night awakening due to wheezing. Approximately one third of the AADC children reported ever having asthma, a physician assessed asthma diagnosis, or being on inhalation steroid treatment. The corresponding frequencies among the ODC children were 9%, 8% and 6%.

**Table 2:** Questionnaire responses: Responses to some of the ISAAC asthma and supplementary questions in the questionnaire.

	Asthma-allergy day-care centres		Ordinary day-care centres	
	
	n	%	n	%
Ever wheezing	454	45.7	1326	27.3
Possibly false croup	5	0.5	58	1.2
Wheezing in last 12 months	385	38.8	908	18.7
1–3 times	170	17.1	656	13.5
4–12 times	139	14.0	193	4.0
> 12 times	74	7.5	49	1.0
Wheezing with no cold	207	20.9	299	6.2
Wheezing at exercise	227	22.9	306	6.3
Severe wheezing* ^)^	105	10.6	134	2.8
Night awakenings** ^)^	286	28.9	564	11.6
< once a week	203	20.5	435	9.0
weekly or more often	83	8.4	129	2.7
Dry cough at night*** ^)^	291	29.5	711	14.7
Ever had asthma	328	33.0	446	9.2
Physician diagnosis	304	30.7	364	7.5
On inhalation steroids	276	27.6	274	5.6
Emergency treatment	206	20.8	354	7.3

*^) ^Wheezing severe enough to interfere with speech

**^) ^Night awakenings due to wheezing

***^) ^Dry cough at night with no cold

### Potential diagnostic criteria

The criteria asthma diagnosed by a physician and having current symptoms, being on inhalation steroid therapy, ever had asthma and having current symptoms, experienced four or more wheezing episodes during the last 12 months, and experienced any wheezing during the last 12 months, in ODC children were chosen for further analysis. As shown in Table 3, the four first criteria or criteria combinations gave fairly similar age and sex-specific asthma prevalence levels. The fifth criterion, any wheezing during the last 12 months, usually not used as single asthma criterion in studies of preschool children, gave a 2–3-fold higher prevalence.

**Table 3:** Asthma prevalence by criteria : Asthma prevalence at ordinary day-care centres (model 1) using five potential diagnostic criteria.

		Prevalence of diagnostic criteria											
		
		Boys aged						Girls aged					
		
		1	2	3	4	5	6	1	2	3	4	5	6
N		112	485	503	506	583	287	90	432	472	512	585	319
1	Physician diagnosis and any wheezing last 12 months	8.0	7.9	8.6	8.5	6.2	6.0	1.1	4.9	6.0	5.7	4.7	2.5
2	Being on inhalation steroids	8.0	6.6	6.6	7.9	5.5	5.9	0	3.7	5.3	6.1	5.0	3.1
3	Ever had asthma and any wheezing last 12 months	8.0	9.2	8.6	9.2	6.4	6.4	1.1	6.3	6.8	6.3	5.0	3.2
4	Wheezing ≥ 4 times last 12 months	10.7	8.1	6.4	5.4	3.8	5.3	2.3	5.6	3.0	4.1	3.3	2.5
5	Any wheezing last 12 month	34.8	28.1	22.9	18.8	13.5	14.1	19.1	22.0	16.6	14.1	12.9	8.2

**Figure 4:**
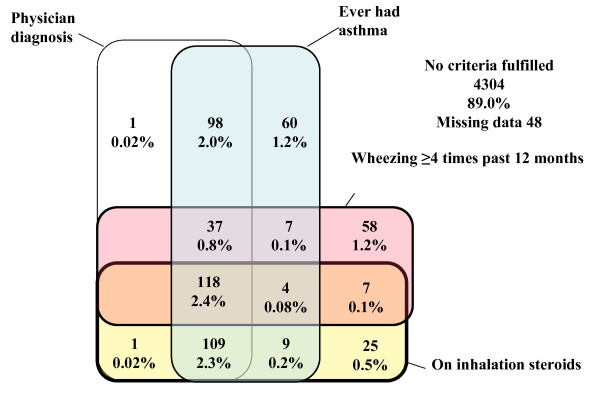
**Diagnostic criteria overlap**. Venn diagram showing overlap between the four diagnostic asthma criteria asthma diagnosed by a physician, ever had asthma, four or more wheezing episodes during the past 12 months, and being on inhalation steroid therapy in ordinary day-care centre children 1–6 years old. Percentages refer to number of children with criterion or criteria combination in relation to total ordinary day-care centre study population.

The overlap between the four first criteria is depicted in Figure 4. Generally, there was a considerable overlap among all criteria. The most common criteria or criterion combinations were all four criteria combined (2.4% of all ODC children), the combination physician diagnosis, ever had asthma and being on inhalation steroids (2.3%) and the combination physician diagnosis and ever had asthma (2%). Other combinations and single criteria were infrequent.

### Prevalence calculations

A combination of diagnostic criteria 1 or 2 or 3 or 4 in Table 3 yielded an asthma prevalence across all sex and age groups of 9.0% (model 1). There were no significant asthma prevalence differences between response batches. The age and sex-specific prevalence based on model 1 and smoothed with logistic regression technique is shown in Figure 2. The prevalence among boys fell linearly across age while it was curvilinear among girls. According to model 2 the shape of the relation between prevalence and age was curvilinear and similar for boys and girls, but boys had on average 1.5 per cent units higher prevalence than girls and the maximum prevalence occurred somewhat later, Figure 3. For boys, the prevalence was 9.7% at age 1, reached a maximum of 11.4% at age 3 and then fell to 6.4% at age 6. The corresponding prevalence levels for girls were 8.9%, 9.8% (at age 2–3), and 5.0%. The mean prevalence, irrespective of age, was 9.6% for boys and 8.2% for girls, altogether 8.9%.

### Asthma prevalence determinants

Based on the prevalence calculation by municipality according to model 2 an analysis of the influence of municipality population size, population density and geographical location on the asthma prevalence adjusted for municipality differences in age and sex distribution was made. Municipality population size, as well as population size at the municipality part where the DCs were located, was tested. Both variables gave the same result. For this reason municipality population size was used in the analyses.

In a first, preliminary age and sex adjusted logistic regression analysis with asthma prevalence as the dependent variable, municipality population density caught 98.5% (Wald's chi-square 598.9) of the demographic variable impact on asthma prevalence whereas municipality population size caught 1.5% (Wald's chi-square 9.1). For this reason population density was used in further analyses. As determined from model 1 parental education had a marginal negative impact (chi-square test, p < 0.05) on asthma prevalence, whereas smoking in the child's home during the first year of life or during pregnancy had no significant effect. For this reason these variables were not used in the final analysis model.

**Figure 5:**
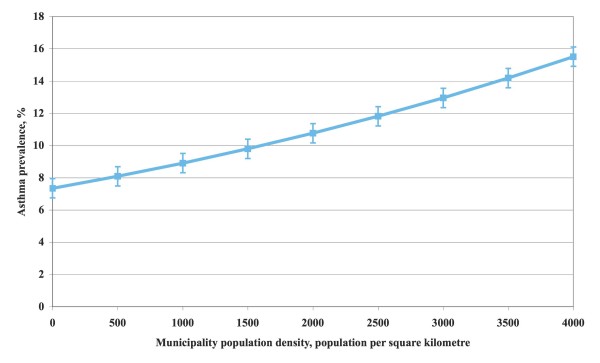
**Asthma prevalence by population density**. Relationship between asthma prevalence in Swedish preschool children 1–6 years old and municipality population density in their home municipality, expressed as number of residents regardless of age per square kilometre municipality area.

The result of the final determinant analysis is shown in Table 4. The asthma prevalence increase by 2% for each 100 residents per municipality square kilometre area, was 19% more common in boys than girls, increased by 7% for each degree north and decreased by 3% for each degree east. The strongest determinants, as measured by Wald's chi-square, were in rank order municipality population density, age, sex, latitude, and longitude. Population density had nearly twice as large impact than all the other determinants together. As shown in Figure 5 asthma prevalence increased in a slowly accelerating pace with population density. Latitude and longitude were still highly significant but had a fairly limited importance over and above that of population density.

**Table 4:** Asthma prevalence determinants : Effects of age, sex, municipality population density, and geographical location (latitude and longitude) on asthma prevalence in Sweden in multivariate logistic regression analysis.

	Odds ratio	95%CI	Wald's chi-square	p
Municipality population density*^)^	1.02	1.02-1.02	1720.6	< 0.0001
Age, years	1.39	1.33–1.44	271.3	< 0.0001
Age squared	0.94	0.94–0.95	456.7	< 0.0001
Male sex	1.19	1.16–1.23	159.7	< 0.0001
Latitude, degree North	1.07	1.06–1.09	147.3	< 0.0001
Longitude, degree East	0.97	0.96–0.98	30.7	< 0.0001

*^) ^Number of residents, divided by 100, per square kilometre municipality area.

## Discussion

In this nationwide study of preschool children the diagnostic criteria commonly used for asthma in childhood all produced fairly similar prevalence levels, as did criteria combinations. Of the two models used to estimate asthma prevalence, model 2, based on adjustment for municipality size and thereby being the most reliable, yielded fairly similar age-specific prevalence for boys and girls. However, boys had on average 1.5 percent units higher age-specific prevalence levels than girls. The strongest asthma prevalence determinant in this study was municipality population density, whereas latitude and longitude had less importance.

The study was based on a large sample of preschool children covering all of Sweden. A possible source of bias might have been the sampling frame, for instance that children attending DCs were more or less healthy than non-attending children. Koopman et al. found that children attending DC had more physician diagnosed lower respiratory tract infections during their first year of life than children cared for at home [25]. Nafstad et al. found no differences in wheeze, chest tightness or current asthma between four-year-old children cared for in DCs or at home [26]. Hägerhed-Engman et al. found a somewhat higher prevalence of wheezing among 1-to-4-year-old children in day-care centres as compared with children cared for at home, but there were no differences in older children and no significant difference regarding physician diagnosis [27]. The most comparable variable in their and our data was having a physician diagnosis. Based on their differences in physician diagnosis between children in DC and children care at home, the physician diagnosis proportion in all children in our data set, whether in DC or at home, would have been 7.1% versus the observed 7.5% among DC children.

Another potential bias might have been the non-random sampling procedure. However, more than 20% of the Swedish municipalities, covering the whole populated part of the country and covering a wide range of population density were included in the study. As pointed out in the Statistical considerations section, the results based on model 1, i.e., based on the study population data analysed straightforwardly, is probably not representative for Swedish children in general. However, the results based on model 2, adjusted for municipality population distribution, are approximately equivalent to those from a random sample, as shown by results from other studies. The potential bias due to the sampling frame is therefore probably small.

The response rate (68%) was satisfactory. The possible bias caused by non-response may be estimated based on the assumptions that non-respondents had asthma diagnosis criteria on average either five standard error units more often or five standard error units less often then the respondents, i.e., a considerable difference. In model 1 the overall asthma prevalence in respondents and non-respondents combined would then have been 9.7% if non-respondents had higher prevalence than respondents, and 8.3% if they had lower prevalence, as compared with the 9.0% we found among respondents. The corresponding prevalence levels in model 2 were 9.0%, 8.8% and 8.9%. The potential bias due to non-response is therefore small.

Our findings indicate that the prevalence of asthma in childhood increases until approximately three years of age and then decreases gradually until age 6. A number of studies have investigated the asthma prevalence in childhood, but none has presented age and sex specific prevalence data for each year in the 1–6 year age range. Bornehag et al. presented age specific data for boys and girls combined, showing the same trends as in this study [6]. Caudri et al. showed trends similar to our model 1 among children in the Netherlands [9]. The BAMSE study reported 8.5% prevalence among 4-year-old boys and 5.3% among girls [28]. The BMHE study reported prevalence in 4-year-old children of approximately 9% among boys and 6% among girls [13]. A number of other studies have reported prevalence among 6–8 year old children with estimates similar to our 6-year estimates [3,29].

Prevalence across age should, however, be interpreted with caution, since there may be secular trends, or cohort effects, involved. However, Swedish ISAAC data indicate that only small changes have occurred over time in specific age groups, indicating small secular effects [5]. The sex and age specific asthma prevalence shown in this report indicates either that a considerable part of asthma in childhood disappears before age 7, as shown by others, or that some asthma cases in reality are attacks of wheezy bronchitis (transient wheezing), that cannot be distinguished from asthma other than by following the course [9,30,31]. However, given the similarities of our results with those of other studies [6,9,13,28] and the fairly low prevalence among the youngest children, where the diagnostic problems are largest, the bias due to the latter is probably moderate to small.

We found highly significant geographical gradients with increasing prevalence towards the north and the west. However, the importance of these gradients was to a large extent attenuated by municipality population density, indicating that a substantial part of the importance of geographical gradients was linked to population density. This is a novel finding not reported before. BMHE reported increasing prevalence towards the north for 12-year-old children but no east-west gradient was presented [13]. Forsberg et al. reported on childhood asthma in four regions in Scandinavia but found no significant difference between suburban and rural areas [14], but in other parts of the world urban-rural prevalence differences have been found [16,17]. As far as we know no previous study in this field has used population density as a preschool asthma prevalence determinant.

Population density was in this study used as a proxy for degree of urbanisation. Population size and density were strongly correlated. We tested not only municipality population size but also population size in the part of the municipality where the DCs were located. Both variables gave similar results. However, municipality sub districts are troublesome to handle because they are not legally defined units in the same way as municipalities, and their borders and areas are thereby defined less strictly than those of the municipalities, resulting in less analysis precision. For these reasons we decided to use only municipality based data.

The results could not be explained by differences in parental education or smoking habits. They could neither be explained by climate, since the population density gradient does not follow the latitude or longitude very closely. However, an association has been shown between population density or degree of urbanisation on the one hand and air pollution or traffic flow density on the other [32-34]. Degree of urbanisation may also coincide with other degree of urbanisation related factors.

There was a residual importance of latitude and longitude when the importance of population density was accounted for. This residual importance might reflect climate factors, such as temperature and humidity. The climate in northern Sweden is sub arctic, whereas the climate in the south resembles that of northern continental Europe. The east-west gradient might reflect air humidity, with more humidity in the west than in the east. However, Weiland et al. found a negative effect on asthma symptom prevalence of altitude, annual temperature variation, and outdoor relative humidity, and no relationship to latitude [12]. De Marco et al. found similar results regarding outdoor temperature [32].

The strengths of the present study include that it was based on a large sample with a national coverage, that well-known instruments were used, and that the attrition rate was moderate and appears to have had little effect on the results. The weaknesses include that the sample was not strictly random, that although the study has a longitudinal nature the results in this report were based on the baseline survey and therefore cross-sectional, and that the results, like those in most other similar studies, were based on questionnaire data only with no access to medical examination data.

## Conclusion

In conclusion, we found that the diagnostic criteria commonly used for asthma in childhood all produced fairly similar prevalence levels, as did criteria combinations. Of the two models used to compute age and sex-specific asthma prevalence, the model based on adjustment for municipality population size yielded fairly similar prevalence by age in boys and girls. However, boys had on average 1.5 per cent units higher age specific prevalence levels than girls. The most important asthma prevalence determinant was population density, as a proxy for degree of urbanisation, catching up more than twice the importance of all other determinants combined. Geographical location affected asthma prevalence only modestly.

## Competing interests

The authors declare that they have no competing interests.

## Authors' contributions

KB, DN, ME, CS, and KS participated in the design of the study. KB and KS performed the analyses. KB, DN, ME, CS, and KS participated in the discussions of the results. KB and KS drafted the manuscript, and DN, ME, and CS participated in the revisions. All authors have seen and approved the final version.

## Pre-publication history

The pre-publication history for this paper can be accessed here:


